# Successful treatment of endotracheal intubation-related lip pressure injury using a self-developed fixation device

**DOI:** 10.1186/s13054-023-04577-5

**Published:** 2023-07-24

**Authors:** Qibing Zhang, Xiaodong Zhang, Chunrong Han, Jiqin You, Yunxia Zhao, Rong Xu, Shiyan Zhao, Yan Geng

**Affiliations:** 1 Nanjing Lishui People’s Hospitial (Zhongda Hospital Lishui Branch), Southeast University, Nanjing, 211200 China; 2 The Central Hospital of Xuzhou, Xuzhou, 221000 China

Dear editor,

Orotracheal intubation is the most common method for establishing artificial airways and plays a significant role in rescuing critically ill patients [1]. However, orotracheal intubation is associated with several complications, including iatrogenic mucocutaneous damage, catheter dislodgement, and tooth loosening [1, 2]. These complications may result in prolonged hospitalisation, increased infection opportunities, and burden the healthcare system and society [2, 3]. Therefore, proper endotracheal tube fixation is essential for preventing complications in patients undergoing orotracheal intubation. Herein, we report the successful treatment of a 79-year-old patient with lip pressure injury using a novel self-developed mask endotracheal tube fixation device (utility model patent of China, ZL 2022 2 1745523.5; Fig. 1A). The fixation device comprises a triangular headband, base, connecting bridge, fixation nut, and bite block (Fig. 1B). The base comprises a mask frame and silicone pad. The connecting bridge is printed using a photosensitive resin. The fixation nut is adapted from a traditional endotracheal tube fixator.

**Fig. 1:**
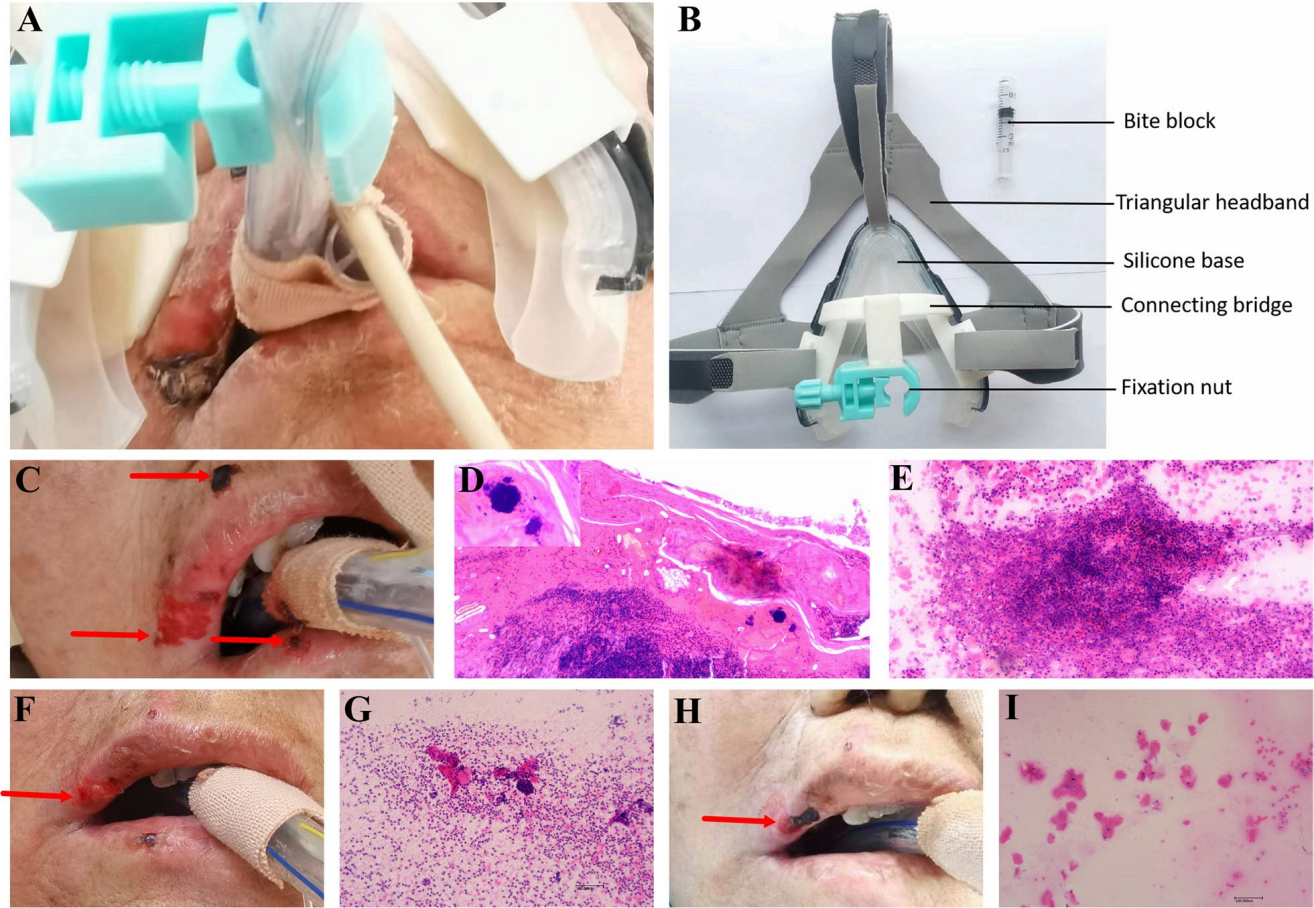
**A** Photograph of the patient wearing the self-developed endotracheal tube fixation device. **B** Structure diagram of the self-developed mask endotracheal tube fixation device. **C** Lip pressure injuries before application of the self-developed mask endotracheal tube fixation device. **D** Histopathological image of damaged lip tissue before application of the self-developed mask endotracheal tube fixation device (the enlarged part in the image showed bacterial colonies). **E** Cytopathological image of damaged lip tissue before application of the self-developed mask endotracheal tube fixation device. **F** Slightly improved lip pressure injuries on the third day after device replacement. **G** Cytopathological image of damaged lip tissue on the third day after device replacement. **H** Significantly improved lip pressure injuries after device replacement. **I** Cytopathological image of damaged lip tissue after device replacement

A 79-year-old Chinese woman was admitted to an intensive care unit with dyspnoea. She had a history of diabetes mellitus, hypertension, and cerebral infarction. Using a traditional endotracheal tube fixator, orotracheal intubation connected to a ventilator for assisted breathing, and oral care was provided every 6 h. Six days later, three pressure injuries were observed on her lips and the surrounding regions (Fig. 1C). Pathological examination of tissue samples revealed epidermal fractures, focal pseudoepithelioma-like hyperplasia, dermal vascular expansion, acute and chronic inflammatory cell infiltration in the epidermis and dermis, parakeratosis of the shed squamous epithelial cell mass, bacterial colonies in the inflammatory exudative, and necrotic tissue (Fig. 1D). Additionally, tissue-print cytology indicated inflammatory exudation, necrosis, and large amounts of red blood cells (Fig. 1E). Sustained pressure above a threshold can cause prolonged ischaemia and lead to tissue necrosis [4]. The pressure wounds were likely initially colonised by bacterial flora and that a bacterial imbalance resulted in a bacterial infection [3]. Bacterial infections impede wound healing, especially in diabetic patients [5]. To prevent further lip pressure injury and considering the patient’s history of diabetes mellitus, we replaced the traditional endotracheal tube fixator with a self-developed fixation device. After explaining the procedure to the patient’s husband, we placed the self-developed fixation device. On the third day after device replacement, the ruptured regions surrounding the right upper lip was smaller than those earlier (Fig. 1F). Moreover, tissue-print cytology showed decreased inflammatory exudation and necrosis (Fig. 1G). On the fifth day after device replacement, the upper edge of the lip peak and the vermilion border of the right lower lip were completely healed, and the ruptured regions surrounding the right upper lip was significantly smaller (Fig. 1H). Tissue-print cytology indicated a significant reduction in inflammatory exudation and necrosis, without any bacterial colonies or repaired epithelial cells (Fig. 1I). The endotracheal tube did not dislodge, and no new pressure injury occurred while the self-developed fixation device was in place.

To the best of our knowledge, this is the first report of a patient with lip pressure injury treated with this novel endotracheal tube fixation device combined with histopathological analyses. Compared with the traditional endotracheal tube fixator, our new device has two advantages. First, it avoids the perioral tissues, improves the labial blood circulation, and promotes the repair of pressure injury. Second, it increases the contact area on the face, thus reducing the pressure per unit area and preventing pressure injuries. Our report introduces a novel endotracheal tube fixation device that is safe and useful for nursing patients undergoing orotracheal intubation.

## Data Availability

The datasets used and/or analysed during the current study are available from the corresponding author on reasonable request.
